# Zoonotic Pathogens of Dromedary Camels in Kenya: A Systematised Review

**DOI:** 10.3390/vetsci7030103

**Published:** 2020-08-05

**Authors:** Ellen Clare Hughes, Neil Euan Anderson

**Affiliations:** 1 The Royal (Dick) School of Veterinary Studies and the Roslin Institute, University of Edinburgh, Roslin EH25 9RG, UK; Neil.Anderson@ed.ac.uk; 2 Institute of Biodiversity, Animal Health and Comparative Medicine, University of Glasgow, Henry Wellcome Building, Garscube Campus, Glasgow G61 1QH, UK

**Keywords:** dromedary, camel, zoonoses, Kenya, systematic review

## Abstract

Kenya is home to Africa’s third largest population of dromedary camels, and production at commercial and local levels are increasingly important. In pastoral and nomadic communities in the arid and semi-arid lands (ASALs), camels play a vital role in food security, while commercial milk production and formalized export markets are rapidly emerging as camel populations expand into non-traditional areas. Until recently, little focus was placed on camels as hosts of zoonotic disease, but the emergence of Middle Eastern respiratory coronavirus (MERS-CoV) in 2012, and the discovery of exposure to the virus in Kenyan camels, highlighted the need for further understanding of this area. This systematised review utilised a robust search strategy to assess the occurrence of camel-associated zoonoses in Kenya and to evaluate the quality of the published literature. Seventy-four studies were identified, covering sixteen pathogens, with an increasing number of good quality studies in recent years. Despite this, the area remains under-researched and there is a lack of robust, high-quality research. Trypanosome spp., *Echinococcus granulosus* and *Brucella* spp. appeared most frequently in the literature. Pathogens with the highest reported prevalence were MERS-CoV (0–100%), *Echinococcus granulosa* (7–60%) and Rift Valley fever virus (7–57%). Exposure to *Brucella* spp., *Coxiella burnetii* and Crimean-Congo haemorrhagic fever virus showed higher levels in camel or camel-associated vectors than other livestock species, although brucellosis was the only disease for which there was robust evidence linking camel and human exposure. Zoonotic agents with less severe human health outcomes, such as *Dermatophilosus congolensis* and contagious ecthyma, were also represented in the literature. This review provides an important summary of the scope and quality of current knowledge. It demonstrates that further research, and improved adherence to robust study design and reporting are essential if the zoonotic risk from camels in Kenya, and elsewhere, is to be better understood.

## 1. Introduction

Kenya is home to Africa’s third largest population of one-humped, dromedary camels (*Camelus dromedarius*) and they account for approximately 5% of the country’s livestock [1]. Camel production has long played a vital role in nomadic and pastoral communities, but formalised production, aimed primarily at lucrative urban milk markets, as well as a thriving international export market, are increasingly important [2,3,4,5]. Camels are unique amongst livestock species in their ability to thrive in arid environments, providing an important source of food and financial security to vulnerable communities, particularly in the face of climate instability [6,7,8]. Camel keeping in Kenya was traditionally focussed in pastoral communities in the arid and semi-arid lands (ASALs) in the northern and north-eastern regions, but as interest in camel production and awareness of their value in food security has developed, populations have expanded into non-traditional areas such as Isiolo and Laikipia; a move supported by the Kenyan government’s ‘2030 vision’ [7,9,10].

Perhaps due to their capacity to thrive in harsh environments, camels were previously considered resistant to many diseases common in other production animals [11,12]. However, the expansion of camel production has led to a re-evaluation of traditional assumptions regarding the species’ susceptibility to disease [13,14], and the emergence of Middle Eastern respiratory syndrome coronavirus (MERS-CoV) in Saudi Arabia in 2012 brought their potential as a zoonotic reservoir into sharp focus [15,16]. Zoonotic pathogens, both in Kenya and globally, disproportionally affect the poorest communities, who tend to live in closer proximity to livestock and often have limited access to medical and veterinary services [17,18]. The benefits of a One Health approach to zoonotic disease control, particularly in poor and isolated communities, has become well established in Kenya over the last decade, but research has tended to focus on cattle and small ruminants [19,20]. As camel production expands, opportunities for zoonotic transmission events are likely to increase. Developing a better understanding of potential zoonotic hazards is an important first step towards reducing the frequency and impact of these events. In light of these concerns, a systematic evaluation of the literature was undertaken to assess the occurrence of zoonotic pathogens associated with dromedary camels in Kenya, and the scope of the published literature. The aims of the review were to evaluate the scope and quality of the literature, as well as to collate prevalence and strain-typing data, with a view to identifying gaps in current knowledge and priorities for future research.

## 2. Materials and Methods

### 2.1. Search Strategy and Record Assessment

A systematised review of the literature was carried out, taking into consideration the guidelines set out in the PRISMA statement, and by adapting best-practice guidelines and protocols developed by other systematic review reporting systems [21,22,23,24,25,26]. Nine databases and collections relating to medical and veterinary disease, global health and basic science were searched to provide a comprehensive assessment of published literature (CAB abstracts, Global Health, Medline, PubMed, Web of Science, BIOSIS, EMBASE, Zoological Record and Africa-Wide Information). Searches covered publications up to the end of December 2017. Grey literature, including media articles and unpublished government reports were excluded due to difficulties in verifying their contents, and comprehensively searching for them.

Zoonotic infections of camels deemed likely to be of relevance in Kenya were determined through review of camel health and production literature, literature relating to zoonoses of other livestock species in East Africa, and the Kenyan Government Zoonotic Disease Unit’s list of priority zoonoses [27]. Only pathogens that could cause clinical disease in humans and be transmitted via human/camel contact, close association such as via aerosol or fomite spread, or by vector transmission were included. This excluded most food-borne pathogens including *Escherichia coli* and *Salmonella* species.

Search terms defining the population, location and disease exposure were combined using the Boolean operator “AND”, and Boolean syntax was adapted to the requirements of the different databases. Terms relating to population included “camel/camels” and “dromedary/dromedaries”. Location terms used were “Kenya” and “Kenyan.” For disease terms, “zoono*” was combined using the Boolean operator ‘OR’ with terms relating to individual zoonotic diseases of camels. *Trypanosoma* species (spp.) were included due to recent evidence of the zoonotic potential of *Trypanosoma evansi* [28,29,30]. Population, location and disease searches were combined using the Boolean operator “AND”. A full list of diseases identified, and the basic search terms used are presented in Table 1. Search terms and inclusion criteria were reviewed by both authors prior to commencement of searches, and review was undertaken by the first author only.

Citations were compiled in EndNote^TM^ and duplicate entries removed. Remaining titles and abstracts were subject to three levels of review: (i) title and abstract review, (ii) full-text review and (iii) quality review. At each stage, citations, abstract or full-text papers were included or excluded according to predefined criteria (Supplementary Materials, Figures S1 and S2). Data were extracted from the remaining full-text citations using a standard data extraction form and the details entered into Microsoft Excel (2016). Where citations were published in more than one location, the more recent or more complete citation was selected. During the full-text review, reference lists of relevant papers were searched by hand and additional papers added.

### 2.2. Quality Criteria Assessment

The quality criteria set out in Alonso et al. [24] were used to assess the level of bias in the full-text review papers. An adapted version of the Alonso et al. criteria is presented in Table 2**.** All papers, regardless of quality designation were included in the qualitative review and discussion. For the purposes of presenting quantitative data on prevalence, only data from papers deemed to be of medium or good quality were reported [24].

## 3. Results

### 3.1. Summary

**Figure 1:**
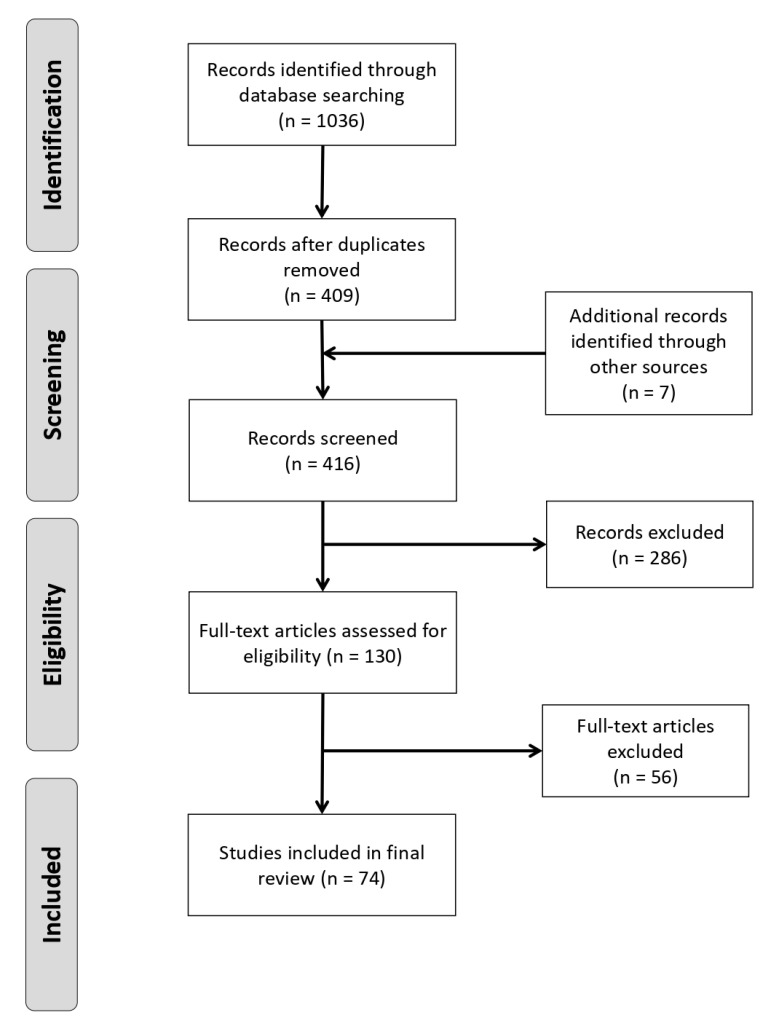
PRISMA flowchart showing numbers of studies at each stage of the review [31].

Following the three stages of review, 74 unique studies were identified as fitting all pre-defined criteria (Supplementary Materials, Figures S1 and S2). The PRISMA flow diagram, showing the numbers of references identified and removed at each stage, is shown in Figure 1.

**Figure 2:**
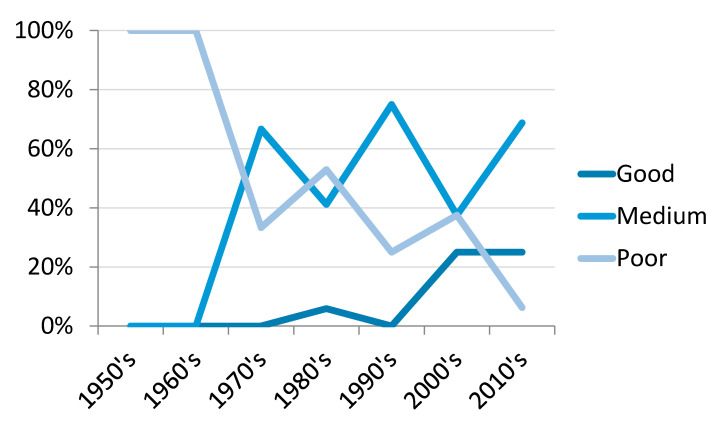
Percentage of total studies per decade categorised as good, medium or poor quality based on the criteria set out by Alonso et al. [24].

Twenty-four papers (32.4%) were deemed to be of poor quality, 41 (55.4%) medium and nine (12.2%) of good quality. All but one of the good quality studies were published since 2000, with a trend towards an increasing proportion of good or medium quality publications and a decrease in poor quality studies over the course of the review (Figure 2).

**Figure 3:**
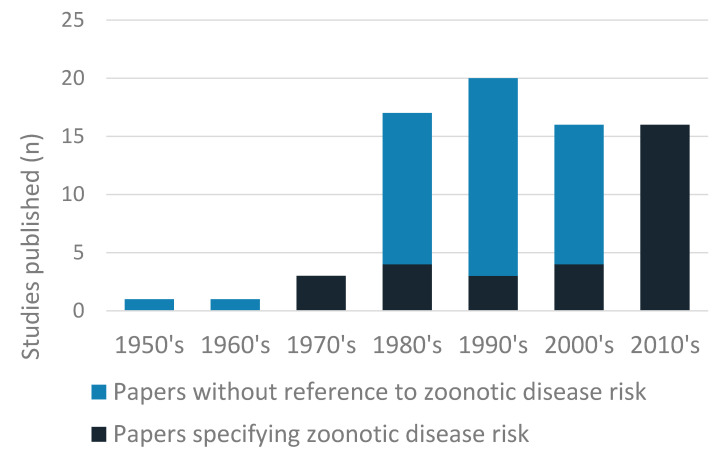
Total number of studies published by decade, showing the number of publications that highlighted the zoonotic risk of the pathogen and those that did not.

Sixteen pathogens, or genera of pathogens, were identified: thirteen in camel hosts, four in ticks retrieved from camels, and two camel-specific strains of pathogen identified in humans (Table 3). Eight viruses, five bacteria, one protozoa, one fungus and one endoparasite were identified. The largest number of papers (*n* = 29, 39.2%) dealt with trypanosome species, with the next most frequently reported pathogens being *Echinococcus* spp. (*n* = 9, 12.2%) and *Brucella* spp. (*n* = 7, 9.5%). Five studies were published before 1980, with between 16 and 20 studies published in each decade since. Zoonotic potential was specifically mentioned in 29 papers (39.1%) and an increase in such studies was observed since 2010, with all papers since this date highlighting zoonotic risk (Figure 3). Forty-two studies dealt with disease surveillance (56.8%), with the majority of these employing a cross-sectional design to determine prevalence.

**Figure 4:**
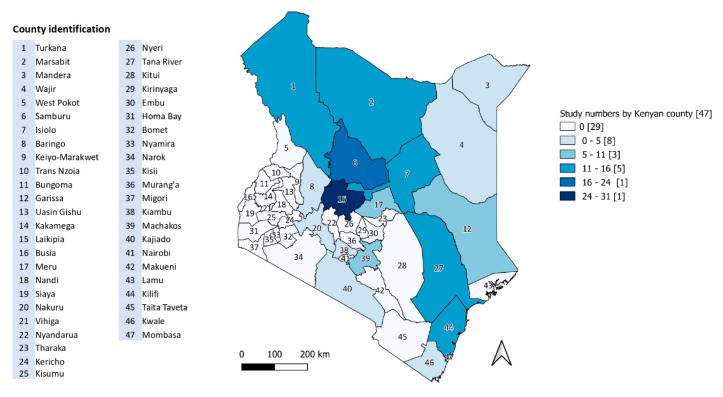
Number of published studies on zoonotic pathogens of camels in Kenya by county.

Prevalence figures reported in medium- and good-quality papers are reproduced in Table 4. Strain typing and pathogen characterisation was the focus of 17 (23.0%) studies and diagnostic test development or validation accounted for 13 (17.6%) publications. The remaining studies included treatment trials, risk evaluations, disease impact and disease outbreak investigations or case studies. Half of identified papers (*n* = 37) referred to a county level location. Figure 4 shows the number of studies by county.

### 3.2. Viruses

Half the zoonotic hazards identified were viruses, with MERS-CoV, Rift Valley fever (RVF) virus (RVFV) and camelpox the most commonly described (Table 3). MERS-CoV was the focus of four eligible good- and medium-quality papers between 2014 and 2017. All publications described cross-sectional studies to determine current or historic serological exposure to MERS-CoV in camels or humans, with the most recent study examining exposure in linked camel and human populations [33]. Prevalence estimates in camels ranged from 6.1% to 100% depending on diagnostic method, age and location [33,34,35]. Liljander et al. found 0.18% of humans tested positive for MERS-CoV specific antibodies, but no humans sampled in the linked household study were positive by the same diagnostic method (Table 4) [32,33]. RVF was also the focus of four papers, two of poor and two of medium quality (Table 3). Early evidence indicated that RVFV was the cause of camel abortions during the 1961–1962 epidemic, confirming the presence of the virus in the arid northern counties [38,39]. Both medium-quality papers demonstrated high seroprevalence during the 2006–2007 epidemic, with Britch et al. [37] also reporting a pre-epidemic prevalence of approximately 7% (Table 4). Camelpox was the focus of four studies, with two of medium quality. One reported a cross-sectional survey (Table 4), and one employed a variety of laboratory techniques to characterise two strains of Kenyan camelpox [40,41]. No studies have been published on camelpox in Kenya since 1997 [40]. Contagious ecthyma, caused by a parapox virus [103,104], was reported in camels in two medium quality studies. One reported on an outbreak of clinical disease in Laikipia in 1984 [47], while the other presented results of a cross-sectional survey in Turkana (Table 4) [46].

Crimean-Congo haemorrhagic fever virus (CCHFV) was the focus of two studies, both of medium quality. One found evidence of exposure in camels imported from Kenya to Egypt [45] (Table 4), while the second used reverse transcription PCR (RT-PCR) to screen ticks collected from livestock in north-eastern Kenya in 2008 [44,45]. Five tick pools were positive for CCHFV (1.4%) [44]. Morrill. et al. also tested samples for antigenically related Nairoviruses, including Dugbe virus [105], which was also detected in ticks from camels in Garissa and Isiolo in a survey of tick-borne viruses between 2007 and 2010 [45,48]. Nine percent of tick pools positive for Dugbe virus came from camel hosts. Dhori virus, a zoonotic orthomyxovirus, was identified in ticks pooled from a camel in Isiolo, and Kupe virus, which is of unknown pathogenicity in humans, was detected in the same study [48,106,107]. A single paper investigating the seroprevalence of Influenza D and C viruses (IDV and ICV) in livestock found antibodies to one or other of the viruses in almost all Kenyan camels sampled, with evidence of cross-reactivity between IDV and ICV (Table 4) [49].

### 3.3. Bacteria

Five bacterial pathogens associated with camel hosts were identified (Table 3). *Brucella* spp. were reported in seven eligible papers, covering ten counties, between 1978 and 2015 (Table 3). All studies reported serological evidence of exposure, with a single study examining seropositivity in humans and livestock [108]. Two papers reporting cross-sectional surveillance were of medium or good quality (Table 4) [59,108]. Exposure to *Coxiella burnetii*, the causative agent of Q fever, was the focus of three eligible studies (Table 3). One paper, of poor quality, identified four positive camels in Samburu [52] and two medium quality studies investigated prevalence in camels in Laikipia in 2011 and 2013 [50,51]. Skin conditions caused by *Dermatophilus congolensis* were reported in five studies in Laikipia, Samburu and Turkana counties, one of which was of medium quality [53]. The presence of five *Rickettsia* species in ticks removed from camels was reported in a single cross-sectional study in pastoralist areas of Garissa and Isiolo counties [64]. Sixty percent of positive tick pools were collected from camels, compared to 31% from cattle, 17% from sheep and 14% from goats. A single study, deemed to be of poor quality, indicated the presence of *Mycobacteria* spp. in camels ranched between Tana River and Kilifi Counties [60]. Fifteen camels (36.6%) reacted following the intradermal skin test and acid-fast bacteria were detected on impression smears from a single lung lesion.

### 3.4. Parasites and Fungi

One endoparasite, one protozoa and one fungi were identified in the literature (Table 3). Cystic echinococcus, caused by the dog tapeworm *Echinococcus granulosus* sensu lato (s.l.) was reported in ten studies. Where county location was specified, all but one focussed on Turkana [98]. One study reported only human infection while all other studies reported infection in camels and other livestock species [99]. Eight studies presented strain or genetic typing evidence (Table 5), while cross-sectional disease surveillance data were presented in two publications (Table 4) [98,102]. All studies were of medium or good quality. Studies concerned various species and strains of *E. granulosus* sensu lato. The categorisation of *E. granulosus* has changed considerably over the period of the review and three different categorisation systems are represented in the eligible papers (Table 5). *Trypanosoma* spp. were the focus of the largest number of studies (*n* = 28). All papers investigated the presence of *Trypanosoma brucei*-type (trypanozoon) organisms and 26 confirmed *T. evansi* specifically. Although one study specified a potential zoonotic risk from *T. evansi*, no typanosome species responsible for typical human African trypanosomiasis (HAT) were identified. A single study presented evidence of mixed infections with *Dermatophilus congolensis* and *Trichophyton verrucosum*, a zoonotic dermatophyte fungal pathogen [53].

## 4. Discussion

This review documented evidence of 16 zoonotic pathogens in dromedary camels in Kenya. The pathogens with the highest reported prevalence in camel populations were MERS-CoV, *Echinococcus granulosa* s.l. and RVFV, while *Brucella* spp., *Coxiella burnetii* and CCHFV showed higher levels in camels or camel-associated vectors than other livestock species (Table 4). Brucellosis was the only pathogen for which robust evidence was identified linking camels with increased human disease risk, although a lack of evidence for this link in other pathogens may be due to insufficient research rather than an absence of association [108]. 

### 4.1. Trends in Camel Research

Livestock census and aerial estimates indicate that camel populations have expanded over the last 30 years and the increasing importance of camel production to the Kenyan economy is widely accepted [14,117,118,119]. In contrast, publication numbers of eligible studies remained broadly similar in each decade since the 1980s, indicating that camel zoonoses research in Kenya has not increased in line with population expansion [120,121]. However, the proportion of papers that explicitly referred to zoonotic risk have shown a substantial increase since 2010 (Figure 2), suggesting an increasing importance placed on this dynamic. The proportion of studies characterised as medium or good quality also increased over the period of the review (Figure 2), which may in part reflect the emergence of veterinary epidemiology as a distinct field of study. Prior to the most recent decades, nearly half of identified publications were of poor quality, with small sample sizes, a lack of clear sampling frame, or incomplete reporting of results preventing many studies from providing robust estimates of prevalence, even where these figures were recorded. A propensity towards poor-quality research in zoonotic disease studies has been identified elsewhere, and deserves greater attention at research and government level to improve the quality of camel studies in general, and of camel zoonoses in particular [24]. It is vital that high-quality research is available to enable policy makers and other stakeholders to make appropriate decisions about zoonotic disease priorities, controls and preventions.

### 4.2. Study Locations

The location of studies partly reflects the distribution of camel populations, with more studies focusing on counties in the northern regions of the country (Figure 4). However, research location was dominated by Laikipia, despite it being a non-traditional camel keeping area. The semi-arid county is one of the most important in Kenya for wildlife and has a mixture of large commercial ranches, wildlife conservancy and pastoralist lands [122]. It is easily accessible compared to counties further north and has a history of livestock research and improvements, which may account for its overrepresentation in the literature [50]. In addition, the Laikipia camel population is estimated to have increased by a factor of nearly five between 1982 and 2010 and is likely to keep expanding as it is also home to the country’s only large commercial camel dairy [50]. By contrast, counties such as Mandera and Wajir, which have both the highest camel populations and largest proportions of the population living under the poverty line, are under-represented in the literature [123]. Logistic and security issues may deter researchers from undertaking projects in these areas. Somali pastoralists and other communities in this region may be at greater risk of camel-associated zoonoses due to higher camel densities and poor access to medical and veterinary services. The lack of published research in these areas suggests that those who rely most heavily on camels for their health and livelihoods may be receiving the least benefit from current research.

### 4.3. Viral Zoonoses

MERS-CoV is the most prominent camel associated zoonosis worldwide and has caused more than 800 deaths since it emerged in Saudi Arabia in 2012 [124]. Dromedary camels are the principal reservoir host and several camel to human transmission events have been confirmed in the Middle East [125,126]. The papers identified in this review show strong evidence of high MERS-CoV seroprevalence in camels in northern and north-eastern Kenya and Laikipia County as far back as 1983, suggesting the virus is endemic in the national herd [34,127]. This is consistent with studies from across the continent [111,127,128,129,130]. The first evidence of human exposure to MER-CoV in Sub-Saharan Africa was reported in Tana River County by Liljander et al. in 2016 [32]. The human seroprevalence in this study was similar to that found in Saudi Arabia [112] and may suggest that human clinical cases are going unreported in Kenya. However, the lack of evidence for human seroconversion in households where camel herds had very high seroprevalence found by Munyua et al. in 2017 suggests camel to human transmissibility of MERS-CoV may be lower in Kenya compared to populations with similar camel exposure in the Middle East [33]. The mechanisms for this apparent difference in transmission risk remain unclear and the emergence of MERS-CoV in human populations in Kenya is still of concern [131,132]. 

Rift Valley fever is a significant public health concern and is recognised as one of the top priorities for zoonotic disease research and control in Kenya [27,133]. Evidence of clinical disease and high levels of seroconversion in camels identified by this review suggest that camels may play an important role in amplification and maintenance of the virus [37,38]. Similar findings from other outbreaks across Africa support this theory, and suggest that camels may be particularly sensitive indicators of RVFV activity, in part due to their long-distance movements as well as their apparent sensitivity to the virus [39,134,135,136]. With raised awareness amongst pastoralist camel-keepers it is possible that camel abortion could act as an early warning of RVF virus infection [137]. Investigations into the potential role of camels in viral amplification and as sentinels are currently limited and this gap should be addressed in future studies.

Camelpox virus is reportedly endemic in East Africa [138,139] and several studies that fell outside the inclusion criteria of this review reported clinical outbreaks or high seroprevalence [12,139]. These observations were rarely supported by robust study design or laboratory diagnosis, making assessment of the true burden difficult, while the zoonotic nature of camelpox is poorly understood and rarely cited [41]. No cases of human disease have been recorded in Kenya, although Davies et al. reported unconfirmed descriptions of humans developing ulcers on the lips and mouth following consumption of milk from visibly affected camels [41]. Early literature suggested that zoonotic transmission of the virus was rare and did not present a public health concern [138,140], but more recently, verified human cases have led to a reassessment of its zoonotic potential [141,142,143]. In the post-smallpox world, with the emergence of a strain of zoonotic orthopox virus a possibility [144], further surveillance and epidemiological investigation of camelpox would be wise [141].

Crimean-Congo haemorrhagic fever virus (CCHFV) is a globally under-researched tick-borne virus with a high case-fatality rate in humans and potential for human-human transmission [145,146]. The first human infection in Kenya, a single acute case, was reported in 2002, and in 2012 a study in Ijara (now Garissa County) found a seroprevalence of 19% (95% CI 15–22%) in febrile patients presenting to local health facilities [145,147]. *Hyalomma* spp. ticks are the principal vectors and are found on all major livestock species in Kenya including camels [44,48]. The exact role of different livestock species remains unclear, but CCHFV positive ticks were only obtained from cattle and camels, suggesting these species may be involved in disease transmission and amplification. Camels may be a particularly important focus for research given their potential to transport infected ticks over long distances.

Findings reported by Salem et al. suggest, for the first time, that camels appear to be hosts of Influenza C and possibly Influenza D viruses (ICV and IDV) [49]. ICV is a known human pathogen, typically causing mild disease in young patients and has been found in other mammalian species including pigs and domestic dogs, while the host tropism of IDV is less well understood [148,149,150]. Although cross-reactivity between the two viruses made estimates of true prevalence difficult to determine, the evidence presented by Salem et al. gives reason to further investigate the role of camels in the tropism of these viruses [49].

### 4.4. Bacterial Zoonoses

Brucellosis is one of the most widespread and significant bacterial zoonoses worldwide, causing severe disease in humans and livestock, as well as imposing a substantial economic burden [151,152]. Serological evidence of *Brucella* exposure in camels was first reported in Kenya in 1978 but despite a prevalence of approximately 10%, no further studies investigated the pathogen in camels until 2012 [59,61,63]. The study conducted by Osoro et al. in 2015 is worthy of note as the only paper reviewed to find an explicit link between camel exposure and increased odds of human seroprevalence [108]. Human studies indicate that the burden of disease in camel-producing regions is high. A study in remote hospitals in Garissa and Wajir found that 13.7% febrile patients were positive for *Brucella abortus* and contact with multiple animal species was significantly associated with infection [151]. Evidence of association between humans and animals in the same household demonstrates the value to be gained from a One Health approach, and the association with camel ownership suggests that a potential source of *Brucella* infection may be overlooked in typical studies focusing on cattle and small ruminants [108,153]. Similar studies, investigating how human interactions with camels influence zoonotic disease risk, should be prioritised. The findings of this review also indicate that *Coxiella burnetii*, the causative agent of Q fever, may be another neglected bacterial zoonosis of camels in Kenya. *C. burnetii* exposure is widespread amongst livestock and humans in the country but is poorly understood and under-reported [154]. Only two recent studies have investigated *C. burnetii* in Kenyan camels but the significantly higher camel seroprevalence compared to cattle found by Browne et al. [50] is consistent with findings from elsewhere in Africa [155].

A number of other bacterial pathogens were identified. *Dermatophilus congolensis* causes exudative dermatitis in multiple species and is typically associated with tick and biting fly-transmission, although it can also be spread by contact [156,157,158,159]. Prevalence levels were similar in Kenya to Sudan and Iran, at between 12 and 30%, and were significantly higher during the wet season [53,160,161]. This may indicate an increased risk as camel production moves into areas with higher rainfall. Human cases are reported sporadically in the medical literature, but none have been reported in Kenya [157,162,163]. The typically self-limiting presentation of the disease in humans is likely to mean that cases go unreported. However, disease can be more serious and debilitating in certain individuals, so health professionals working with livestock keepers should remain vigilant [164]. *Rickettsia* spp. were identified from various species of ticks in Garissa and Isiolo counties and although full numbers were not reported, significantly more *Rickettsia* positive tick pools came from camel-associated ticks than other livestock hosts [64]. Despite evidence of circulation of several zoonotic *Rickettsia* spp. in Kenya, few acute human clinical cases have been recorded [165,166,167]. However, the lack of distinct clinical features as well poor access to laboratory diagnostics means febrile illness caused by *Rickettsia* spp. is likely under-reported [64,168]. Further surveillance is required to develop a fuller picture of the true prevalence and range of *Rickettsia* spp. in camel ecto-parasite populations, and the impact of these on human disease risk. The identification of mycobacteria infections in camels, although only identified in one study [60], may be of public health importance as *Mycobacterium bovis* cases have been reported in camels elsewhere in Africa and nomadic communities in Kenya appear to have higher levels of tuberculosis than the general population [169,170]. Although the reasons behind these high infection rates are poorly understood, it is hypothesised to relate to consumption of infected milk. Given the nutritional and economic importance of camel milk for many Kenyan pastoralists, further research on the presence of zoonotic *Mycobacterium* species in camels, particularly in these vulnerable communities, is recommended [118,119].

### 4.5. Parasitic Zoonoses

Extensive research into cystic echinococcosis (CE), caused by species of the canine tapeworm *Echinococcus granulosa* s.l., has been undertaken in Kenya, and studies in this review demonstrate the presence of *E. granulosa* s.l. and *E. canadensis* in camel populations in Turkana, Meru and Isiolo counties. Studies tended to focus on strain typing and cyst viability rather than prevalence due to a lack of reliable and cost-effective diagnostic tests for screening large numbers of animals under field conditions [102,171]. Human infections, both worldwide and in Kenya, are dominated by genotype type G1 (‘common sheep strain’), now categorized as *E. granulosus* sensu stricto (s.s.) [172]. Although prevalence levels appear to be high in Kenya and neighbouring countries, the dominance of the ‘G6/G7’ genotype (now re-categorized as a distinct species, *Echinococcus canadensis*) in camel populations may suggest that this species plays a lesser role in human CE [93,173,174]. However, a review of 1661 human cases globally found that 11% of these were caused by ‘G6/G7’ genotype, and a study of animal and human cases in Sudan found that *E. canadensis* was the dominant cause of human infections [175,176]. These findings suggest that *E. canadensis* may play a more important role in human infection in Kenya than is currently recognised. Historically, research focussed almost exclusively on Turkana County, where unusually high levels of CE are found in the human population [177,178]. Prevalence of *Echinococcus* spp. in camels in Turkana was comparable to that seen in other species, but a slaughter slab study in Isiolo and Meru counties, where levels of CE in the human population are much lower, found camels to have higher levels of infection compared to cattle, sheep and goats [98,102]. The dominance of Turkana as a focus of research may mean that the causes and dynamics of infection in other areas of the country are overlooked.

The greatest number of eligible studies dealt with *Trypanosoma* spp., either identified specifically as *T. evansi* or more generally as *T. brucei*-type trypanosomes, which in camel hosts are most likely to be *T. evansi*. Trypanosomiasis, or Surra, caused by *T. evansi* is considered one of the most important production diseases of camels in East Africa [80,179] but camels are not known to be hosts of *T. brucei gambiense* or *T. brucei rhodesiense*, the species responsible for HAT. Trypanosomes were included in this review due to the recently highlighted zoonotic potential of *T. evansi* but only one study made explicit reference to this [68]. This is consistent with the assumption, until recently, that *T. evansi* was not zoonotic. The first reported case of zoonotic *T. evansi* infection occurred in an Indian farmer in 2004 [180] and since then individual cases have been reported in Egypt and Vietnam [30,181]. Factors affecting the transmissibility of *T. evansi* to humans are not fully understood and the risk is likely to be very low [182]. However, given the importance of *T. evansi* as a production disease of camels, it is possible that occasional cases of human *T. evansi* infections occur in Kenya, but go unreported, and it’s zoonotic potential should not be ignored [29,183].

### 4.6. ‘Missing’ Pathogens

Several zoonotic pathogens of camels were notable by their absence. Sarcoptic mange, caused by the mite *Sarcoptes scabii*, has been listed as an important disease of camels in East Africa and was ranked as one of the top 15 priority zoonoses in Kenya, but no eligible papers referred to this pathogen [10,133]. Cases of mange were described in several studies but these were not included because the diagnosis was based on clinical examination only and not confirmed with appropriate laboratory methods, or because results were incompletely reported [10,88,92]. Studies utilising more robust diagnostic methods would help to characterise this disease risk. No publications were identified relating to *Toxoplasma gondii*, which is known to be an important disease of camels elsewhere and is an under-researched but likely important public health concern in Kenya [184]. A review and meta-analysis of toxoplasmosis in meat animals in Africa found camels to have the highest average prevalence at 36% (95% CI 18–56%) [185], and a study in Ethiopia found a prevalence in camels of 68.2% (95% CI: 63.5% to 72.9%) with the presence of cats or wild felids significantly associated with camel seropositivity [186]. With camel populations increasing in wildlife areas such as Laikipia, further research is needed to determine the significance of this pathogen. A recent publication identified for the first time a prion disease of dromedary camels in Algeria [187]. Although the distribution, and infectious and zoonotic risk of this pathogen is unknown, this discovery provides further evidence of the importance of enhanced surveillance and research into camel disease.

### 4.7. Limitations

The current review has certain limitations, in light of which the results should be viewed. The exclusion of food-borne pathogens omitted a number of important camel-associated zoonoses which would benefit from a comprehensive review, particularly in light of raw camel milk consumption practiced in some pastoral communities [188,189]. Although efforts were taken to construct comprehensive and replicable searches of the published literature, the exclusion of unpublished reports and other grey literature may mean instances of disease outbreaks have been missed. Efforts were made to standardize the quality review process by employing the methods set out in Alonso et al., but subjectivity in this assessment may have introduced a reporting bias [24,190]. To limit bias introduced from poor quality studies, prevalence estimates were only reproduced from medium- or good-quality papers. Although serology is an important tool in disease surveillance, seropositivity may not reflect current infection status or transmissibility of the pathogen. In addition, the number of identified studies does not necessarily reflect the pathogens with the highest prevalence in camels or those that pose the greatest zoonotic risk. No further statistical analysis of the reported data were undertaken, so it was not possible to draw conclusions about camel associated risk beyond those presented by individual publications. Despite these limitations, the evidence identified by this review provides a starting point for further research aimed at quantifying the risk to human populations from camel-associated zoonoses in Kenya.

## 5. Conclusions

The quality findings of this review and the imbalance of research focus are reflective of neglected tropical diseases on a wider scale, whose neglect is often driven by under-reporting and under-estimation of true burden. Good-quality, robust studies on the prevalence, incidence or typing of zoonotic pathogens in camels were limited, and it is vital that camel and zoonotic disease researchers make robust study design and reporting a priority if data are to be useful for the broad interpretation required to inform policy. Where prevalence data were robustly reported, MERS was not the only pathogen to which camels were highly exposed, and these pathogens, as well as those to which camels appeared more exposed than other livestock species, point to priorities for further research. The number of studies which considered camel and human disease together was extremely limited, a situation also reflected in literature on other species in Kenya [191]. It remains to be seen whether the increasing interest in One Health approaches to zoonotic disease research and control will increase the proportion of such studies in future. 

## Figures and Tables

**Table 1:** Search terms and synonyms used to construct searches relating to zoonotic pathogens of camels in Kenya.

Zoonotic Infections/Agents of Relevance	Search Terms and Synonyms (* Indicates Wildcard Search Function)
*Brucella* spp.	“brucel *”
Camelpox	“camelpox”, “pox” “poxvir *”, “orthopox *”
Crimean-Congo haemorrhagic fever virus	“Crimean-Congo haemorrhagic fever *”
*Echinococcus granulosa* sensu lato	“echinococ *”, “* hydatid *”
Emerging pathogens	“emerging” AND “infection *” OR “virus *” OR “bacteria *”
Middle Eastern respiratory syndrome virus	“Middle East respiratory”, “Middle Eastern respiratory”, “MERS”, “MERS-CoV”, “coronavir *”
Q fever (*Coxiella burnetii*)	“q fever *”, “coxiell *”
Rift Valley fever virus	“Rift Valley fever”, “RVF”
Sarcoptes	“sarcopt *”, “mange *”
Toxoplasmosis	“toxoplasma *”
*Trypanosome* spp.	“* trypanos *”, “African trypan *”
Tuberculosis	“TB”, “tubercul *”, “mycobact *”

Wildcard functions (denoted by an *) were used to expand the search terms where appropriate. Search terms and wildcard functions were adapted as required for each database.

**Table 2:** Quality criteria tool used to assess the level of bias in studies selected for inclusion in the review. Adapted from [24].

Good Quality (Low Risk of Bias)	Medium Quality (Moderate Risk of Bias)	Poor Quality (High Risk of Bias)
Unbiased selection of subjects, evidence of randomisation	Bias in subject selection is acknowledged and accounted for or is unavoidable	Bias in subject selection is not acknowledge or accounted for
Appropriate data analysis	Data analysis limitations are acknowledged	Inappropriate data analysis
Scientifically sound methods	Methods are sound but may not be the most appropriate	Methods are inappropriate
Accurately described methods	Methods are comprehensible and valid even if details are lacking	Methods are unclear or incomplete
Accurate and complete reporting of results	Results are reported accurately	Results are inaccurate or incomplete

**Table 3:** Zoonotic pathogens of dromedary camels in Kenya: number of studies identified in this review and host species in which it was identified.

Pathogen or Disease	Number of Studies	Host or Vector of the Pathogen Identified			References		
					Quality Score		
		Camel	Tick †	Human ‡	Good	Medium	Poor
**Viruses**							
MERS-CoV	4	X		X	[32,33]	[34,35]	
Rift Valley fever virus	4	X				[36,37]	[38,39]
Camelpox	4	X				[40,41]	[42,43]
Crimea-Congo haemorrhagic fever virus	2	X	X			[44,45]	
Contagious ecthyma	2	X				[46,47]	
Dugbe virus	1		X			[48]	
Dhori virus	1		X			[48]	
Influenza viruses (ICV and IDV)	1	X				[49]	
**Bacteria**							
*Coxiella burnetii* (Q fever)	3	X				[50,51]	[52]
*Dermatophilus congolensis*	5	X				[53]	[54,55,56,57]
*Brucella* spp.	7	X			[58]	[59]	[12,60,61,62,63]
*Mycobacterium* spp.	1	X					[60]
*Rickettsia* spp.	1		X			[64]	
**Parasites and Fungi**							
*Trypanosoma* spp.	28	X			[65,66,67,68,69]	[10,70,71,72,73,74,75,76,77,78,79,80,81,82,83]	[84,85,86,87,88,89,90,91,92]
*Echinococcus* spp.	10	X		X	[93]	[94,95,96,97,98,99,100,101,102]	
*Trichophyton verrucosum*	1	X				[53]	

† Ticks removed from Dromedary camel hosts only. ‡ Evidence of transmission from camels to human, or of a camel-specific strain of pathogen in human hosts.

**Table 4:** Prevalence values reported in disease surveillance studies categorised as good or medium quality, based on criteria set out in Alonso et al. 2016 [24].

Type	Pathogen	Species	Dates Sampled	Test Used	Number Tested	County or Region	Prevalence % (95% CI) ^†^	Quality	Reference
Virus	Middle Eastern respiratory coronavirus (MERS-CoV)	Camel	1992–2013	Recombinant MERS-CoV spike protein subunit 1-based ELISA (rELISA) described by Memish et al., 2014 [109]	162	North-eastern region	56.2	Medium	[34]
					154	Eastern region	17–100		
					458	Rift Valley region	0–18		
		Camels	2013	Spike protein subunit 1 protein microarray [110,111]	335	Laikipia county	46.9	Medium	[35]
		Camels	2013	AntiMERS-CoV Camel IgG ELISA kit (EUROIMMUN AG, Lübeck, Germany)	879	Marsabit county	90 (95% CI 88–92)	Good	[33]
		Humans		AntiMERS-CoV Camel IgG ELISA kit (EUROIMMUN AG, Lübeck, Germany) followed by plaque reduction neutralisation test (PRNT) [112]	760	Marsabit county	0		
		Humans	2013–2014	rELISA (EUROIMMUN AG, Lübeck, Germany) followed by PRNT [112]	559	Garissa county	0	Good	[32]
					563	Tana River county	0.36		
	Crimean-Congo haemorrhagic fever virus	Camels	1986–1987	Agar gel diffusion (AGD) test [113]	499	Not specified	26	Medium	[45]
	Contagious ecthyma	Camels	Not specified	Clinical examination and electron microscopy	600	Turkana	11.2	Medium	[46]
	Rift Valley fever virus	Camels	2006–2007 (epidemic period)	In-house IgG ELISA [114]	110	Not specified	20.9	Medium	[36]
		Camels	2000 (pre-epidemic period)	In-house inhibition ELISA [115]	15	Galana county	6.7	Medium	[37]
					13	Garissa county	7.7		
			2007 (epidemic period)		28	Isiolo county	57.1		
	Camelpox	Camels	1992	Clinical examination, electron microscopy, virus neutralisation	1000	Samburu county	27	Medium	[40]
					1200	Turkana county	6		
	Influenza D virus (IDV)	Camels	2015	Hemagglutination inhibition (HI), post-ICV hemadsorption	293	Not specified	8.2	Medium	[49]
	Influenza C virus (ICV)	Camels	2015	HI, post-IDV hemadsorption	293	Not specified	10.6	Medium	[49]
Bacteria	*Brucella* spp.	Camels	Not specified	Rose Bengal plate test (RBPT)	174	Warir, Garissa and Mandera counties	4.6	Medium	[59]
				Serum agglutination test (SAT)			10.34		
				Complement fixation test (CFT)			9.77		
		Camels	2013	Brucella-Ab C-ELISA kit (SVANOVIR, Uppsala, Sweden)	1605	Marsabit county	11.1 (95% CI 9.4–15.0)	Good	[108]
	*Coxiella burnetii* (Q fever)	Camels	2011	ELISA CHEKIT Q fever test kit (IDEXX, Westbrook, ME, USA	72	Laikipia county	Adults (3–9 years) 46 Young (<6 m) 5	Medium	[50]
		Camels	2013	ELISA CHEKIT Q fever test kit (IDEXX, Hoofddorp, The Netherlands)	334	Laikipia county	19	Medium	[51]
	*Dermatophilus congolensis*	Camels	1993	Clinical examination and bacterial isolation	3200	Samburu county	Wet season, 20.9 Dry season, 13.6	Medium	[53]
					600	Laikipia county	Wet season, 22.7 Dry season, 14.3		
Parasites	*Echinococcus* spp.	Camels	1998–2000	Post-mortem examinations	70	Turkana county	60.1	Medium	[102]
		Camels	2013	Post-mortem examination and RFLP-PCR [98]	219	Meru and Isiolo counties	6.94	Medium	[98]
	*Trypanosoma* spp.	Camels	1996–1997	Haematocrit centrifugation technique (HCT) Mouse inoculation test (MIT) Suratex^®^ latex agglutination test (Brentec Diagnostics, Nairobi, Kenya) [71]	103	Athi River (Machakos county)	2.9 (95% CI 0–6.2)	Medium	[73]
					749	Isiolo county	25.4 (95% CI 22.3–28.5)		
					86	Mugwoni (Laikipia county)	18.6 (95% CI 10.4–26.8)		
		Camels	Not specified	Phase contrast buffy coat technique (BCT) MIT	347	Kajiado county	33.8	Medium	[10]

^†^ 95% confidence intervals (95% CI) only reproduced here if reported in the original study.

**Table 5:** *Echinococcus granulosus* sensu lato characterised according to the nomenclature set out in each paper to describe strain, species and genotype, with host species and diagnostic method reported.

*Echinococcus granulosus* Species/Genotype	Host Species	Method of Confirmation	County/Location	Reference
*E. granulosus* Type B	Camel	Electrophoresis: isoelectric focusing	Turkana	[97]
*E. granulosus* Type A	Human	Electrophoresis: isoelectric focusing	Turkana	[97]
*E. granulosus* ‘Common sheep strain’	Camel	PCR and electrophoresis	Turkana	[100]
*E. granulosus* ‘Camel strain’	Camel	PCR and electrophoresis	Turkana	[100]
*E. granulosus* G1	Camel Humans	PCR	Turkana/Maasai	[116]
*E. granulosus* G6 (G6/7)	Camel Human	PCR	Turkana/Maasai	[116]
*E. granulosus* G1	Human	PCR	Turkana	[99]
*E. granulosus* G6	Human	PCR	Turkana	[99]
*E. granulosus* sensu stricto (s.s.)	Camel	PCR	Meru/Isiolo	[98]
*E. canadensis* (formally G7)	Camel	PCR	Meru/Isiolo	[98]
*E. Canadensis* G6/7 cluster	Camels Human	PCR	Not specified	[93]

## Supplementary Materials

The following are available online at https://www.mdpi.com/2306-7381/7/3/103/s1, Figure S1: Title and abstract inclusion/exclusion protocol, Figure S2: Full-text inclusion/exclusion criteria.
