# Supplementary material for: Zoonotic Pathogens of Dromedary Camels in Kenya: A Systematised Review
Source: Vet Sci. 2020 Aug 5;7(3):103. doi: 10.3390/vetsci7030103 (PMC7559378; doi:10.3390/vetsci7030103)
Supplement: Supplementary file 1 [file vetsci-07-00103-s001.pdf]

Figure S1

Title and abstract inclusion/exclusion protocol

1. Study population
    a. Include if:
        i. Dromedary or camel included in study
    b. Exclude if:
        i. Only species other than Dromedary camel
2. Geographic location
    a. Include if:
        i. Presents data from Kenya
        ii. Presents data from several countries, including Kenya
        iii. Presents data on biological samples of Kenyan origin
    b. Exclude if:
        i. Presents data only from countries other than Kenya
3. Pathogen
    a. Include if:
        i. Potentially zoonotic pathogen of camels transmitted via contact, vector, aerosol or fomites
    b. Exclude if:
        i. Food-borne or other orally transmitted pathogen or commensal organism of camels which may cause human disease
4. Date
    a. Include if:
        i. Published up to 31 December 2017
5. Article type
    a. Include if:
        i. Peer-reviewed journal article, conference proceedings, PhD or Master's Thesis
    b. Exclude if:
        i. Review article not presenting original data
        ii. Textbooks and clinical guides not presenting original data
        iii. Newspaper articles and other lay media publications
6. Study type
    a. Include:
        i. Cross-sectional studies
        ii. Case-control studies
        iii. Cohort studies
        iv. Retrospective or prospective study designs
        v. Case studies
        vi. Treatment trials undertaken in naturally infected camels
        vii. Diagnostic test studies using naturally infected camels
        viii. Studies presenting data related to molecular or genetic characterisation of naturally occurring infections
        ix. Studies that present ethno-graphic or socio-economic data as well as original biological data

b. Exclude:

    i. Experimental study design, including in vitro and in vivo studies examining cellular, molecular or other aspects of infection not occurring in naturally infected animals
    ii. Studies dealing with physiological response to infections in naturally infected animals
    iii. Laboratory methods descriptions and reviews which do not provide data on naturally occurring infections in Kenya
    iv. Diagnostic test validation studies which do not present data on naturally occurring infection in Kenya
    v. Studies evaluating physiological, cellular or other responses to treatment
    vi. Studies presenting ethnographic data on camel diseases without original biological data
    vii. Studies concerned with other socio-economic aspects of camel disease without presenting original biological data
    viii. Studies which presented evidence of suspected diagnosis not confirmed by laboratory testing

References for which no abstract is available and those which do not conclusively meet the inclusion/exclusion criteria after title/abstract review should be carried forward for full-text analysis.

Figure S2

Full text inclusion/exclusion criteria

The above criteria are also applied to the full text review, with the following additional conditions:

1. Language
    a. Include if:
        i. Full text available in English
    b. Exclude if:
        i. Full text not available in English
        ii. Abstract only available in English, full text only available in language other than English
2. Article type
    a. Include if:
        i. Full text available
    b. Exclude if:
        i. Abstract only published
        ii. Conference proceeding without full text available
3. Study population
    a. Exclude if:
        i. Population already under treatment for pathogen of interest
4. Study type
    a. Exclude if:
        i. Treatment or diagnostic test studies which does not present evidence of prevalence or incidence of infection in population prior to treatment
        ii. Studies reporting development of molecular techniques (e.g. PCR etc) which do not also provide epidemiological information on natural infections in Kenya
        iii. Phylogenetic studies which do not also provide epidemiological information regarding natural infections in Ken
